# Association between Experience of Pet Ownership and Psychological Health among Socially Isolated and Non-Isolated Older Adults

**DOI:** 10.3390/ani11030595

**Published:** 2021-02-24

**Authors:** Tomoko Ikeuchi, Yu Taniguchi, Takumi Abe, Satoshi Seino, Chiho Shimada, Akihiko Kitamura, Shoji Shinkai

**Affiliations:** 1 Human Care Research Team, Tokyo Metropolitan Institute of Gerontology, Tokyo 173-0015, Japan; shimadac@tmig.or.jp; 2 Center for Health and Environmental Risk Research, National Institute for Environmental Studies, Tsukuba 305-0053, Japan; taniguchi.yu@nies.go.jp; 3 Integrated Research Initiative for Living Well with Dementia, Tokyo Metropolitan Institute of Gerontology, Tokyo 173-0015, Japan; abe@tmig.or.jp; 4 Research Team for Social Participation and Community Health, Tokyo Metropolitan Institute of Gerontology, Tokyo 173-0015, Japan; seino@tmig.or.jp (S.S.); kitamura@tmig.or.jp (A.K.); 5 Kagawa Nutrition University, Saitama 350-0288, Japan; shinkai.shoji@eiyo.ac.jp

**Keywords:** dog ownership, cat ownership, social isolation, psychological health, older adult

## Abstract

**Simple Summary:**

The COVID-19 pandemic may have accelerated social isolation, particularly of older adults who are at increased risk of severe illness, while practicing physical distancing and self-quarantine. This study conducted in a metropolitan area of Japan hopes to provide stronger cross-cultural evidence of the positive impact of pet ownership on the psychological health of socially isolated owners with its large sample of older adults comparing two types of pet ownership (i.e., dog or cat). In this study, experience of dog or cat ownership and the presence or absence of social isolation were categorized into four groups to compare psychological health of each group. After adjusting for demographic and potential confounders, we found that socially isolated older adults who never owned a dog were more likely to report lower psychological health in comparison to socially isolated current or past dog owners. Our findings have practical implications that pets, particularly dogs, can play a role in increasing opportunities for engaging in physical and social activities and providing emotional support and thereby reducing a sense of social isolation and loneliness and improving psychological health among older adults.

**Abstract:**

The psychological health effects of pet ownership have been widely studied, but only a few studies investigated its impact among socially isolated older adults. The present study aims to investigate the psychological health of older adults with or without the experience of pet (i.e., dog or cat) ownership who are socially isolated or not socially isolated. This study used cross-sectional data from 9856 community-dwelling older adults in a metropolitan area of Japan. Social and non-social isolation and type of pet ownership (i.e., dog or cat) were stratified to examine the psychological health. Logistic regression models indicated that, after adjusting for demographic and potential confounders, socially isolated older adults who never owned a dog were 1.22 times more likely to report lower psychological health in comparison to socially isolated current or past dog owners. No such difference was observed among cat owners. The results suggest that the experience of dog ownership may be effective to improve the psychological health among socially isolated older adult.

## 1. Introduction

Along with population aging and decreasing fertility in most industrialized countries, the proportion of single and childless adults has been increasing, and more people are growing older while living alone. In Japan, for example, the percentage of one-person households among those aged 65 and older increased from 10.7% in 1980 to 27.4% in 2018 [1]. Living alone is one primary risk factor associated with social isolation [2]. A nationally representative study conducted in Japan found that 30% of older men living alone had no one to ask for even a little help in their daily lives [3]. Social isolation in later life is linked to various adverse psychological and health-related outcomes, such as cardiovascular impairment, chronic pain, loneliness, and depression [4,5,6,7,8,9].

The coronavirus disease 2019 (COVID-19) pandemic, which has impacted the physical and psychological health of millions of people worldwide [10,11], has required older adults to self-isolate by remaining at home and avoiding social contact with family and friends [12]. While the COVID-19 pandemic may have accelerated social isolation, particularly of those living alone, some pet owners have benefitted from their pets during this period of physical distancing and self-isolation. A study conducted in Australia during the COVID-19 lockdown revealed that dog ownership was a significant protection against loneliness for adults living alone [13]. Positive outcomes associated with pet ownership have been reported, such as reducing loneliness [14,15,16], lowering levels of depression [17], and decreasing depressive symptoms [18]. Our previous study on pet ownership and living arrangements among older Japanese found that the psychological health of older pet owners living alone was as good as older adults living with family members but without pets [19]. Social arrangements such as pet ownership in later life could represent a type of living arrangement that needs to be better understood.

The psychological health effects of pet ownership have been widely studied. However, only a few studies investigated its impact on socially isolated older adults. Moreover, studies that have been conducted to date reflect inconsistent findings. In one such study, levels of depression in a small sample of homebound older adult pet owners and non-pet owners were examined, but no significant difference was observed [20]. Another study of sexual minority older adults with disability and limited social network found that those living with pets had significantly higher perceived social support associated with positive psychological outcomes than those without pets [21]. In addition, an increasing number of studies have reported varying outcomes among dog owners and cat owners. Hajek and Konig found that single female dog owners were less lonely than single female non-pet owners, but no such result was observed among cat owners and male pet owners [22].

While existing studies suggest that there may be a relationship between pet ownership and the psychological health of their socially isolated owners, the findings to date are inconsistent. This present study conducted in Japan hopes to provide stronger cross-cultural evidence of the positive impact of pet ownership on the psychological health of socially isolated owners with its large sample of older adults comparing two types of pet ownership (i.e., dog or cat). We hypothesized that the experience of pet ownership would be effective to improve the psychological health of socially isolated older adults.

## 2. Materials and Methods

Our present study is based on a large, randomized survey sample of community-dwelling older adults in a metropolitan area with varying levels of socioeconomic status and different types of living arrangements. It examined the interaction between social isolation as measured by levels of psychological health with pet ownership. Due to the mixed findings reported among cat owners and dog owners in previous studies, stratified analysis was conducted for social isolation and the experiences of dog or cat ownership after adjusting for relevant confounders.

### 2.1. Data

This study used baseline data from a study called the Ota Genki Senior Project collected in 2016 in Ota Ward, Tokyo, Japan [23]. Ota Ward is the southernmost of the 23 wards of the Tokyo metropolitan area. In August 2016, the population of Ota Ward was 716,645, and 22.7 percent (162,443) were aged 65 years or older. We mailed self-administered questionnaires to 15,500 non-disabled residents aged 65 to 84 years based on stratified and random sampling strategies in all districts of Ota Ward in 2016. Of those, 11,925 questionnaires were returned, resulting in a response rate of 76.9 percent. The individuals who completed the survey questions related to social interactions, dog or cat ownership, and psychological health were selected for this cross-sectional study (N = 9856). 

### 2.2. Sample Characteristics

The mean age of the Japanese participants was 79.9 (SD 5.5) years and ranged from 65 to 84 years. Half were women (50.1%). Thirty-eight percent reported graduating from high school, and 36.3% reported attending at least some college. The median household income was between 1,000,000 yen and 2,500,000 yen (equivalent to 9,500 and 23,810 U.S. dollars). More than 93% of the participants reported being able to perform the instrumental activities of daily living, such as transporting, shopping, and managing finances. Overall, 19.6% were living alone, and 32.0% were socially isolated. Among those living alone, 25.3% were socially isolated, whereas 29.7% of those living with others were socially isolated. Nearly a third of the participants (27.7%) were identified as having low levels of psychological health. For pet ownership, 14.1% were current dog or cat owners, 29.7% were past owners, and 56.2% never owned a dog or cat. Among all pet owners, the socially isolated dog or cat owners only represented 3.7% of the total. By comparison, 8.3% were socially isolated past owners, and 20.0% of the socially isolated never owned a pet. Stratified by dog ownership, 31.8% were current or past dog owners, 68.2% were never dog owners. Likewise, 17.5% were current or past cat owners, and 82.5% were never cat owners. Only 0.1% reported owning both a dog and a cat. 

### 2.3. Psychological Health

Psychological health was assessed using the Japanese version of the World Health Organization- Five Well-Being Index (WHO-5), a self-administered five-item scale. This is a widely used survey tool consisting of 5 questions assessing the degree of positive well-being of the respondents during the past two weeks. The items include: (1) ”I have felt cheerful and in good spirits,“ (2) ”I have felt calm and relaxed,” (3) “I have felt active and vigorous,” (4) “I woke up feeling fresh and rested,” and (5) “My daily life has been filled with things that interest me” [24]. Each item is scored from 5 (all of the time) to 0 (none of the time), and the total score ranges from 0 to 25, with higher scores representing greater psychological health, which can be considered as the aggregate of satisfaction with life. Low scores on the WHO-5 may indicate a clinical depression [24]. The present study dichotomized psychological health with scores over 13 as high and those below 13 as low. A cut-off point of <13 yielded the best sensitivity/specificity trade-off in the screening of depression by Awata et al. [25]. The validity of the WHO-5 has been confirmed in samples of Japanese older adults [26].

### 2.4. Dog and Cat Ownership

Participants were asked about their experience with pet ownership, and respondents were categorized as either current, past, or as having had no experience with pet ownership. Those with current or past pet ownership experience were asked about the type of pet in the household (i.e., dog, cat, or other). Since the questionnaire did not include the length nor the timing of pet owning experience, those current and past pet owners were combined for analysis.

### 2.5. Social Isolation

Social isolation was measured using four-question items of social interactions that are: “How often do you see or go out with your friends or neighbors,” “How often do you talk to your friends or neighbors by phone,” “How often do you talk to your family members or relatives who do not live with you,” and “How often do you talk to your family members or relatives who do not live with you by phone.” Participants answered the frequency of these interactions by selecting from the following eight items that apply: (1) every day, (2) every other day, (3) every two or three days, (4) once a week, (5) two or three times a month, (6) once a month, (7) less than once a month, or (8) none. We defined socially isolated older adults as those who answered once a week or less for all four questions.

### 2.6. Ethical Considerations

The Ota Genki Senior Project was carried out following the relevant guidelines of the Ethics Committee of the Tokyo Metropolitan Institute of Gerontology. We adhered strictly to the Declaration of Helsinki [27]. This study was approved in June 2016 by the Ethics Committee of the Tokyo Metropolitan Institute of Gerontology. A statement attached to the questionnaire explained the purpose of the survey, the voluntary nature of participation, and a promise of anonymity in the analysis. Returning the questionnaire was considered as consent to participate in the study.

### 2.7. Statistical Analysis

Initially, a stratified analysis of social isolation with experience of dog or cat ownership was conducted. Subsequently, we examined the independent associations of social isolation and the experience of dog or cat ownership with psychological health, after adjusting for demographic and potential confounders using logistic regression analysis. Potential confounders were evaluated for collinearity and included age, sex, living arrangements (living alone or not living alone), income level, and living districts of the study area. 

Statistical analyses were conducted using SPSS (version 26.0, Armonk, NY). A *p*-value of less than 0.05 was considered as indicative of statistical significance.

## 3. Results

Demographic characteristics of socially or non-socially isolated community-dwelling older adults with or without the experience of dog or cat ownership are presented in Table 1 and Table 2. We identified 7.6% as socially isolated current or past dog owners, 21.2% socially isolated never dog owners, 24.2% non-socially isolated current or past dog owners, and 47.0% non-socially isolated never dog owners. For cat owners, 4.4% were socially isolated current or past cat owners, and 24.5% were socially isolated never cat owners, 13.1% were non-socially isolated current or past cat owners, and 58.1% were non-socially isolated never cat owners. 

### Social Isolation and Dog or Cat Ownership on Psychological Health

The associations of socially or non-socially isolated older adults with or without the experience of dog or cat ownership and psychological health are shown in Table 3. A stratified multiple logistic regression analysis adjusted for sex and age, indicated that when comparing with socially isolated current or past dog owners, socially isolated never dog owners were associated with significantly higher odds ratios (ORs) of low psychological health: 1.29 (95% confidence interval [CI]: 1.08–1.53). After adjusting for demographic and potential confounders, socially isolated never dog owners were 1.22 (1.03–1.46) times more likely to report low psychological health, whereas non-socially isolated current or past and never dog owners were associated with significantly lower odds ratios: 0.46 (0.39–0.56) and 0.50 (0.42–0.59) respectively. For cat ownership, logistic regression models yielded an OR of 0.50 (0.39–0.63) in non-socially isolated current or past cat ownership and 0.45 (0.36–0.55) for non-socially isolated never cat owners for low psychological health, as compared with socially isolated current or past cat ownership, after adjusting for demographic and potential confounders. There was no significant difference in psychological health between socially isolated current or past cat owners and socially isolated never cat owners. For both dog and cat owners, the psychological health of socially isolated individuals was lower than that of non-socially isolated individuals. We also added dog and cat ownership separately to each model as a potential covariate, due to the probability that some people own both dogs and cats. However, OR values did not change as noted in Table 3.

## 4. Discussion

This cross-sectional study of 9856 community-dwelling older adults in Japan used stratified analysis and logistic regression models to investigate the psychological health of those with or without the experience of dog or cat ownership who are socially isolated or non-socially isolated. After adjusting for demographic and potential confounders of age, sex, income level, and living arrangements, socially isolated current or past dog owners had better psychological health than socially isolated individuals who were never dog owners. However, there was no such difference observed among current or past cat owners. This is consistent with existing studies [28], pointing out that no empirical evidence has supported cat owners as less lonely than non-cat owners. In contrast, socially isolated dog owners may still find a need to walk their dogs daily and thereby engage in daily physical activities that contributes to their psychological health [29]. Taniguchi et al. also support this point when they stated that extended periods of dog ownership could enhance both the physical and social functions of the owners through dog-walking [30]. Furthermore, studies have shown that dogs are highly capable of communicating with humans by reading human cues and discriminating emotional and facial expressions (e.g., [31,32]). Although those socially isolated dog owners may have limited interactions with humans, dogs can be playing a role in providing emotional support to their owners.

In the present study, current and past pet owners were combined for analysis. One may think that the past experience of pet ownership would not affect the current owners’ psychological health. However, many Japanese pet owners often preserve the bond they felt with their pet even after the pet’s death [33]. It is not unusual to place a deceased animal in a pet cemetery in Japan, visit the grave regularly, make a pet altar at home, and hold memorial services for their pet [34]. We believe that the past experience of pet ownership has the capacity to provide some level of emotional support to the owner even when the pet is no longer there physically. Future studies should investigate how the length and the timing of pet owning experience affect the current psychological health of past pet owners. 

In summary, our research provides additional empirical evidence of pet ownership and dog ownership in particular on reducing the deleterious effects of social isolation on older adults. Our survey of community-dwelling older Japanese found empirical support based on a very large sample of older adults for pet ownership to enhance psychological health and thereby reduce the negative effects of social isolation. The present study represents an important confirmation of the value of human-animal interaction given its analysis of the relationship between dog and cat ownership and social isolation after adjusting for confounding factors. 

The consequences of social isolation among older individuals are significant. Not only is it affecting the health and well-being of older adults, it is also indicative of the way people are dying. Maeda [35], for example, estimated that there were approximately 30,000 “lonely deaths” among people aged 65 and older in Japan. The number of lonely deaths is estimated to have tripled since the year 2000. Lonely deaths or *koritsushi* refer to those living alone who are found dead in their homes without being noticed for days or weeks [36,37]. When older adults living alone are unmotivated to interact with anyone, it is often difficult for public health officials or social workers to approach to offer or provide services. For those socially isolated older adults who are reluctant to engage in human contact, different strategies are needed. If dog companions can increase the opportunities of engaging in physical and social activities, it could be an important method to help older adults break out of social isolation and its associated socio-psychological problems of boredom, depression, physical inactivity (e.g., [38]), and possibly lonely deaths. 

It is worth noting that there is also a concern of animal hoarding among socially isolated older adults living with pets that have been reported on numerous occasions by the Japanese news media (e.g., [39]). There may be a need to build a support system for older adults who wish to live with pets and to assist those owners who are no longer able to care of their pets or themselves. Specifically, during difficult times such as the COVID-19 pandemic, caring for pets can be challenging for the older owners without adequate means of support [40]. Is there a need for pet sharing or pet renting services for those who cannot afford the full cost and responsibility of pet care? Is there a need to consider the value or efficacy of robotic pets as an alternative? These and other social isolation abating strategies may need to be explored further as the older adult population in many industrialized countries rapidly increases, and social isolation compounds the cost of eldercare. 

A few caveats need to be considered when interpreting the findings of our study. First, we assessed the frequency of social interactions to measure social isolation as the objective state of being isolated from others. However, fewer social interactions or smaller social networks do not necessarily relate to lower psychological well-being in old age [41]. Future studies should attempt to evaluate whether those who isolate by choice are less affected by social isolation. Second, this study involves a cross-sectional correlational design comparing people who choose to live with pets with those who do not. If more specific measures regarding health status and wealth were collected, it may have been possible to determine whether people who are in better health and wealth are more likely to have pets and maintain them for extended periods. Third, data on the length of time of pet ownership were not available in our present study. Finally, as related to these caveats above, the specific process between dog or cat ownership and psychological health among socially isolated older adults remains unclear. Hence, our findings cannot discuss the intricacies of the effects of pet companionship (i.e., “pet effect”). Future longitudinal studies are needed to examine the full effects of pet companionship that would be enhanced by the length of pet ownership.

## 5. Conclusions

This is the first study to assess the psychological health among individuals in different social isolation statuses (i.e., socially isolated or non-socially isolated) and pet ownership statuses (i.e., pet owners or non-pet owners), using a very large, randomized sample of community-dwelling older adults in an East Asian metropolitan area. We found that socially isolated current or past dog owners had better psychological health than socially isolated older adults who were never dog owners. Our findings have practical implications that pets, particularly dogs, can play a role in increasing opportunities for engaging in physical and social activities and providing emotional support and thereby reducing a sense of social isolation and loneliness and improving psychological health. Although the implications of this study may be limited to those who can and want to live with pets, our findings may also be practical under conditions such as the COVID-19 pandemic that may have elevated levels of loneliness and social isolation among older adults while practicing “social distancing.”

## Figures and Tables

**Table 1:** Demographic characteristics of 9856 socially and non-socially isolated community-dwelling older adults with or without experience of dog ownership.

Variable	Socially Isolated Current/Past Dog Owners		Socially Isolated Never Dog Owners		Non-Socially Isolated Current/Past Dog Owners		Non-Socially Isolated Never Dog Owners	
Participants, n (%)	746	(7.6)	2093	(21.2)	2389	(24.2)	4628	(47.0)
Age, years								
65–74, n (%)	359	(48.1)	1034	(49.4)	1282	(53.7)	2243	(48.5)
75–84, n (%)	387	(51.9)	1059	(50.6)	1107	(46.3)	2385	(51.5)
Female sex, n (%)	267	(35.8)	741	(35.4)	1356	(56.8)	2578	(55.7)
Living arrangements								
Living alone, n (%)	84	(11.3)	403	(19.3)	371	(15.5)	1069	(23.1)
Not living alone, n (%)	662	(88.7)	1690	(80.7)	2018	(84.5)	3559	(76.9)
Income level ^†^								
<1,000,000 yen, n (%)	60	(8.0)	173	(8.3)	130	(5.4)	314	(6.8)
1,000,000 yen–2,500,000 yen, n (%)	252	(33.8)	850	(40.6)	634	(26.5)	1505	(32.5)
2,500,000 yen–4,000,000 yen, n (%)	182	(24.4)	508	(24.3)	607	(25.4)	1293	(27.9)
≥4,000,000, n (%)	129	(17.3)	264	(12.6)	657	(27.5)	953	(20.6)
Unknown	123	(16.5)	298	(14.2)	361	(15.1)	563	(12.2)
Psychological health								
High (≥13), n (%)	471	(63.1)	1196	(57.1)	1891	(79.2)	3572	(77.2)
Low (<13), n (%)	275	(36.9)	897	(42.9)	498	(20.8)	1056	(22.8)

^†^ The four levels of income are equivalent to <9500.00 U.S. dollars, 9500.00–23,810.00 U.S. dollars, 23,810.00–38,100.00 U.S. dollars, and ≥38,100.00 U.S. dollars (1 U.S. dollar = 105 Japanese yen).

**Table 2:** Demographic characteristics of 9856 socially and non-socially isolated community-dwelling older adults with or without experience of cat ownership.

Variable	Socially Isolated Current/Past Cat Owners		Socially Isolated Never Cat Owners		Non-Socially Isolated Current/Past Cat Owners		Non-Socially Isolated Never Cat Owners	
Participants, n (%)	429	(4.4)	2410	(24.5)	1291	(13.1)	5726	(58.1)
Age, years								
65–74, n (%)	237	(55.2)	1156	(48.0)	671	(52.0)	2854	(49.8)
75–84, n (%)	192	(44.8)	1254	(52.0)	620	(48.0)	2872	(50.2)
Female sex, n (%)	146	(34.0)	862	(35.8)	753	(58.3)	3181	(55.6)
Living arrangements								
Living alone, n (%)	55	(12.8)	432	(17.9)	244	(18.9)	1196	(20.9)
Not living alone, n (%)	374	(87.2)	1978	(82.1)	1047	(81.1)	4530	(79.1)
Income level ^†^								
<1,000,000 yen, n (%)	23	(5.4)	210	(8.7)	82	(6.4)	362	(6.3)
1,000,000 yen–2,500,000 yen, n (%)	154	(35.9)	948	(39.3)	383	(29.7)	1756	(30.7)
2,500,000 yen–4,000,000 yen, n (%)	107	(24.9)	583	(24.2)	351	(27.2)	1549	(27.1)
≥4,000,000, n (%)	81	(18.9)	312	(12.9)	316	(24.5)	1294	(22.6)
Unknown	64	(14.9)	357	(14.8)	159	(12.3)	765	(13.4)
Psychological health								
High (≥13), n (%)	266	(62.0)	1401	(58.1)	991	(76.8)	4472	(78.1)
Low (<13), n (%)	163	(38.0)	1009	(41.9)	300	(23.2)	1254	(21.9)

^†^ The four levels of income are equivalent to <9500.00 U.S. dollars, 9500.00–23,810.00 U.S. dollars, 23,810.00–38,100.00 U.S. dollars, and ≥38,100.00 U.S. dollars (1 U.S. dollar = 105 Japanese yen).

**Table 3:** Independent associations of social isolation and experience of dog/cat ownership with lower psychological health among 9856 community-dwelling older adults.

Independent Variable	Unadjusted	Adjusted for Sex and Age	Adjusted for Sex, Age, Living Situation, Equivalent Income, and Living Districts
	OR (95% CI)	OR (95% CI)	OR (95% CI)
Social isolation & Experience of dog ownership			
Socially isolated current/past dog owners ^§^	1	1	1
Socially isolated never dog owners	1.258 (1.081–1.526) *	1.286 (1.083–1.528) *	1.224 (1.028–1.457) *
Non-socially isolated current/past dog owners	0.451 (0.377–0.539) **	0.443 (0.370–0.530) **	0.463 (0.386–0.556) **
Non-socially isolated never dog owners	0.506 (0.430–0.596) **	0.496 (0.421–0.585) **	0.495 (0.419–0.586) **
Social isolation & Experience of cat ownership			
Socially isolated current/past cat owners ^§^	1	1	1
Socially isolatednever cat owners	1.175 (0.952–1.452)	1.170 (0.947–1.445)	1.110 (0.896–1.374)
Non-socially isolated current/past cat owners	0.494 (0.391–0.624) **	0.482 (0.381–0.609) **	0.496 (0.391–0.629) **
Non-socially isolated never cat owners	0.458 (0.373–0.562) **	0.447 (0.364–0.549) **	0.447 (0.363–0.550) **

* *p* < 0.05, ** p < 0.01. ^§^ Reference group. OR: odds ratio. Psychological health was dichotomized using a score of the WHO-5, and higher ORs indicate lower psychological health. Logistic regression models were run separately.

## Data Availability

The data presented in this study are available on request from the corresponding author. The data are not publicly available due to the sensitivity of the information, and in order to protect the confidentiality of participants.
